# *Fat-1* Transgenic Mice With Augmented n3-Polyunsaturated Fatty Acids Are Protected From Liver Injury Caused by Acute-On-Chronic Ethanol Administration

**DOI:** 10.3389/fphar.2021.711590

**Published:** 2021-08-31

**Authors:** Jeffrey Warner, Josiah Hardesty, Ying Song, Rui Sun, Zhongbin Deng, Raobo Xu, Xinmin Yin, Xiang Zhang, Craig McClain, Dennis Warner, Irina Kirpich

**Affiliations:** ^1^ Division of Gastroenterology, Hepatology, and Nutrition, Department of Medicine, University of Louisville, Louisville, KY, United States; ^2^ Department of Pharmacology and Toxicology, University of Louisville School of Medicine, Louisville, KY, United States; ^3^ James Graham Brown Cancer Center, University of Louisville, Louisville, KY, United States; ^4^ Department of Surgery, University of Louisville, Louisville, KY, United States; ^5^ University of Louisville Alcohol Research Center, University of Louisville School of Medicine, Louisville, KY, United States; ^6^ University of Louisville Hepatobiology & Toxicology Center, University of Louisville School of Medicine, Louisville, KY, United States; ^7^ Department of Chemistry, University of Louisville, Louisville, KY, United States; ^8^ Center for Regulatory and Environmental Analytical Metabolomics, University of Louisville, Louisville, KY, United States; ^9^ Robley Rex Veterans Affairs Medical Center, Louisville, KY, United States

**Keywords:** alcohol-associated liver disease (ALD), fat-1, n3 polyunsaturated fatty acids (n-3PUFAs), plasminogen activator inhibitor (PAI-1), NIAAA model

## Abstract

Alcohol-associated liver disease (ALD) is the leading cause of liver disease worldwide, and alcohol-associated hepatitis (AH), a severe form of ALD, is a major contributor to the mortality and morbidity due to ALD. Many factors modulate susceptibility to ALD development and progression, including nutritional factors such as dietary fatty acids. Recent work from our group and others showed that modulation of dietary or endogenous levels of n6-and n3-polyunsaturated fatty acids (PUFAs) can exacerbate or attenuate experimental ALD, respectively. In the current study, we interrogated the effects of endogenous n3-PUFA enrichment in a mouse model which recapitulates features of early human AH using transgenic *fat-1* mice which endogenously convert n6-PUFAs to n3-PUFAs. Male wild type (WT) and *fat-1* littermates were provided an ethanol (EtOH, 5% v/v)-containing liquid diet for 10 days, then administered a binge of EtOH (5 g/kg) by oral gavage on the 11^th^ day, 9 h prior to sacrifice. In WT mice, EtOH treatment resulted in liver injury as determined by significantly elevated plasma ALT levels, whereas in *fat-1* mice, EtOH caused no increase in this biomarker. Compared to their pair-fed controls, a significant EtOH-mediated increase in liver neutrophil infiltration was observed also in WT, but not *fat-1* mice. The hepatic expression of several cytokines and chemokines, including *Pai-1*, was significantly lower in *fat-1 vs* WT EtOH-challenged mice. Cultured bone marrow-derived macrophages isolated from *fat-1* mice expressed less *Pai-1* and *Cxcl2* (a canonical neutrophil chemoattractant) mRNA compared to WT when stimulated with lipopolysaccharide. Further, we observed decreased pro-inflammatory M1 liver tissue-resident macrophages (Kupffer cells, KCs), as well as increased liver T regulatory cells in *fat-1 vs* WT EtOH-fed mice. Taken together, our data demonstrated protective effects of endogenous n3-PUFA enrichment on liver injury caused by an acute-on-chronic EtOH exposure, a paradigm which recapitulates human AH, suggesting that n3-PUFAs may be a viable nutritional adjuvant therapy for this disease.

## Introduction

Alcohol-associated liver disease (ALD) is a common liver condition resulting from chronic and excessive alcohol consumption. ALD is one of the main causes of liver damage and is the most common reason for liver transplantation in the United States (Seitz et al., 2018). An early manifestation of ALD is hepatic steatosis, an accumulation of fat in the liver that is reversible upon cessation of drinking. However, continued alcohol use may lead to an aberrant and prolonged inflammatory response (steatohepatitis), which may progress to acute alcohol-associated hepatitis (AH) in individuals who consume alcohol in an acute-on-chronic pattern. AH is a severe manifestation of ALD with high mortality and morbidity for which treatment options are limited (Mathurin and Bataller, 2015; Fung and Pyrsopoulos, 2017). Importantly, only a subset of individuals with early stages of ALD will progress to later stages. Susceptibility to ALD is multifactorial and is influenced by patterns of alcohol consumption (Crabb et al., 2020), underlying genetic predisposition (Meroni et al., 2018), obesity (Chiang and McCullough, 2014), and nutrition (McClain et al., 2011; Kirpich et al., 2012; Kirpich et al., 2016; Warner et al., 2017; Zirnheld et al., 2019), among others factors.

Previous work from our group and others demonstrated that modulation of nutritional factors, including dietary and endogenous fatty acids, plays an important role in the pathogenesis of experimental ALD (Kirpich et al., 2012; Huang et al., 2015; Kirpich et al., 2016; Wang et al., 2017; Warner et al., 2017). Specifically, our previous work has focused on the critical role of n3-and n6-polyunsaturated fatty acids (PUFAs) in the development of ALD using preclinical mouse models (Warner et al., 2017; Warner et al., 2018). n3-PUFAs and their metabolites (resolvins, protectins, and maresins) can temper the inflammatory response by decreasing neutrophil infiltration through decreased chemotaxis, adhesion, and trans-endothelial migration (Tull et al., 2009; Dalli et al., 2013). Conversely, n6-PUFAs and their metabolites can promote neutrophil chemotaxis and activate neutrophils leading to increased reactive oxygen species generation (Patterson et al., 2012). Our group showed that mice fed a diet high in n6-PUFAs developed more severe manifestations of ALD than those fed a diet high in saturated fats (Warner et al., 2017). We also demonstrated that n3-PUFA endogenous enrichment, with a concomitant decrease in the n6/n3-PUFA ratio (using *fat-1* mice that endogenously convert n6-PUFAs to n3-PUFAs), attenuated liver damage in an early-stage ALD mouse model characterized by steatosis and modest liver injury (Warner et al., 2019; Hardesty et al., 2021). This protection was afforded by favorable effects on gut barrier function as well as hepatic Wnt signaling (Warner et al., 2019; Hardesty et al., 2021). Similarly, Huang *et al.* demonstrated decreased acute ethanol (EtOH)-induced liver injury and steatosis, as well as decreased lipogenic gene expression, in *fat-1* mice (Huang et al., 2015).

However, the ability of n3-PUFAs to mitigate liver damage in more advanced stages of ALD is largely unexplored. Therefore, in the current study, we investigated the effects of n3-PUFA enrichment in an acute-on-chronic mouse model of ALD that recapitulates more advanced features of human ALD, such as those in early AH, including pronounced liver injury, steatosis, and neutrophil-mediated hepatic inflammation (Jaeschke, 2002; Bertola et al., 2013). We explored the mechanisms leading to the benefits of n3-PUFAs in this context relating to neutrophil infiltration, oxidative stress, and the acute-phase protein PAI-1, which has been shown to be a pathogenic mediator of ALD development (Bergheim et al., 2006).

## Materials and Methods

### Mice and Experimental Design

*Fat-1* transgenic mice that have been engineered to express the *C. elegans* n3-fatty acid desaturase gene (*fat-1*), and therefore have increased tissue n3-PUFAs without the need for dietary intervention, were obtained from J.X. Kang and have been described previously (Kang et al., 2004). These mice were bred in the Association for Assessment and Accreditation of Laboratory Animal Care-accredited animal facility at the University of Louisville, maintained as heterozygotes, and genotyped as previously described (Warner et al., 2019) using primer sequences to the *fat-1* transgene (with *Gdf5* as an internal control, Table 1). Tissue n3-PUFA enrichment and a decrease in the n6/n3-PUFA ratio in *fat-1* mice has been confirmed in our previous reports (Warner et al., 2019; Hardesty et al., 2021), and is consistent with that reported by J.X. Kang for these mice (Kang et al., 2004). 10–12 week old wild type (WT) and *fat-1* ^*+/-*^ male littermates were placed on an all liquid diet that contained 5% (v/v) EtOH for 10 days followed by a single binge of 5 g/kg EtOH on day 11 [the NIAAA, or 10 + 1 model (Bertola et al., 2013),]. Pair-fed (PF) control mice were also provided an all-liquid diet, but with maltose dextrin supplying the same number of calories as EtOH (diets are the Lieber-DeCarli Rodent Liquid Diets (control [PF], F1259SP; EtOH, F1258SP) purchased from Bio-Serv, Flemington, NJ). According to the manufacturer, the PUFA content of both diets was 9.7 g/L, divided between 9.3 g/L linoleic acid (n6) and 0.3 g/L alpha linolenic acid (n3), indicating an n6:n3 ratio of 31:1. Animals were euthanized 9 h following the EtOH binge, and liver tissue was collected and snap-frozen for future analyses. Blood was collected from the inferior vena cava using heparinized 25 g needles, and plasma was separated by centrifugation at 7,000 g for 5 min. Daily food consumption and beginning/ending body weights were recorded. There were four experimental groups in total: WT-PF, WT-EtOH, *fat-1*-PF, and *fat-1*-EtOH (*n* = 8–14 per group). This study was performed under IACUC protocol number 15423 to I.A.K. Note: for simplicity, “*fat-1* ^*+/-*^” mice are referred to as “*fat-1*”.

**TABLE 1:** Primer Sequences.

Gene	Forward (5′-3′)	Reverse (5′-3′)
*fat-1*	CGG​TTT​CTG​CGA​TGG​ATC​CCA​C	CAC​AGG​AAC​CGG​GCA​AAG​AA
Gdf-5	AAG​CCC​TCA​GTC​AGT​TGT​GC	AAA​ACC​ATG​AAA​GGA​GTG​GG
*18S*	CTC​AAC​ACG​GGA​AAC​CTC​AC	CGC​TCC​ACC​AAC​TAA​GAA​CG
*Cxcl2*	GCG​CCC​AGA​CAG​AAG​TCA​TA	TCC​AGG​TCA​GTT​AGC​CTT​GC
*Pai-1*	TCA​ATG​ACT​GGG​TGG​AAA​GG	AGG​CGT​GTC​AGC​TCG​TCT​AC

### Liver Polyunsaturated Fatty Acids Analysis

Liver samples (∼50 mg) were homogenized using water at a ratio of 1 mg sample per 10 µl solvent. 200 µl ethanol was mixed with 200 µl of each homogenized sample to extract the PUFAs. After vortexing, the mixture was centrifugated at 14,000 rpm for 15 min. Solid phase extraction was used to purify and concentrate PUFAs using a Waters Oasis HLB cartridge (1 ml/30 mg). The cartridge was conditioned with 1 ml methanol followed by 1 ml water. After loading all supernatants, the cartridge was washed with 1 ml 5% methanol (v/v). The PUFAs were eluted with 1 ml methanol/acetonitrile (10/90, v/v). The extract was dried by evaporation under a gentle nitrogen gas stream. The dried sample was then redissolved in 75% ethanol for liquid chromatography-mass spectrometry (LC-MS) analysis using a Waters Acquity H-class UPLC system (Milford, MA, United States) coupled with a Waters Xevo TQ-S micro triple quadrupole mass spectrometer (Milford, MA, United States). The chromatographic separation was carried out on a Waters Acquity UPLC BEH C8 2.1 × 100 mm, 1.7 µm column (Milford, MA, United States) equipped with a guard column. The mobile phase consisted of A: 0.1% formic acid in water (v/v) and B: 0.1% formic acid in acetonitrile (v/v). LC-MS conditions are the same as in Yuan et al. (2020). Briefly, the column temperature was held at 40°C. Linear gradient elution was performed at 0.4 ml/min starting at 30% B for 3 min (0–3.0 min), increased to 99% B over 20 min (3.0–20 min), then continued at 99% B over 25 min (20–25 min), and finally returned to 30% B at 25.1 min for column re-equilibration (25.1–28 min). The MS detection was performed using electrospray ionization in negative mode. The relative quantification of each PUFA compound was achieved by multiple reaction monitoring by measuring peak area. Our relative measurement does not allow direct calculation of the n6:n3-PUFA ratio, instead, n3-and n6-PUFAs are reported separately. The data are reported as average fold-change between the two most highly abundant PUFAs for each class (arachidonic acid [AA] and linoleic acid [LA] for n6 and eicosapentaenoic acid [EPA] and docosahexaenoic acid [DHA] for n3) relative to WT PF (set as 1). PUFAs were measured in all mice (*n* = 8–14 per group).

### Assessment of Liver Damage

Plasma alanine aminotransferase activity (ALT, an indirect measure of hepatocyte cell death) was determined using the ALT/GPT reagent as per the manufacturer’s instructions (Thermo Fisher Scientific, Waltham, MA). Liver tissue hematoxylin and eosin (H&E) staining for gross tissue morphology and Oil Red O staining for neutral lipids were performed as described previously (Hardesty et al., 2021). From H&E-stained sections, lipid accumulation was quantified by steatosis scoring, where a score of 0 indicates 0–5% tissue coverage by lipid droplets, 1 = 5–33% coverage, 2 = 33–66% coverage, and 3 = 66–100% coverage (Bedossa and Consortium, 2014). Oil Red O staining was quantitated by capturing 10 random digital photomicrographs, then using a macro in ImageJ to measure the % of the area within the field that was colored red. Total hepatic triglycerides (TGs) were measured using the triglycerides reagent from Thermo Fisher Scientific. Hepatic neutrophils were determined using an ELISA to myeloperoxidase, an enzymatic marker for neutrophils (LifeSpan BioSciences, Inc. Seattle, WA), and by immunohistochemistry with anti-MPO antibodies (R&D Systems, Minneapolis, MN). MPO immunohistochemistry was quantitated by counting MPO+ cells in ten random digital photomicrographs at 200X magnification. The thiobarbituric acid-reactive substances (TBARS) assay was performed on liver tissue to evaluate oxidative stress (Cayman Chemical, Ann Arbor, MI).

### RNA Extraction and Gene Expression Analysis by Semi-quantitative Real-Time PCR

Total RNA from liver samples was purified with Trizol (Thermo Fisher Scientific), contaminating DNA was removed with DNase I (Thermo Fisher Scientific), and cDNAs were synthesized and quantitated using reagents from Quanta Biosciences (Beverly, MA). For gene expression analysis, 10 ng cDNA was assayed (0.01 ng for 18 s) on an Applied Biosystems StepOne real-time PCR instrument (Thermo Fisher Scientific). Data were reduced using the ΔΔCt method (Livak and Schmittgen, 2001). Primer sequences are provided in Table 1.

### Liver Immune Cell Isolation and Flow Cytometry Analysis

Liver immune cell isolation was conducted as previously described (Chu et al., 2020). Briefly, livers were homogenized by mechanical disruption with a rubber-tipped syringe plunger and then passed through a 70 µm strainer to obtain a single cell suspension. Immune cells were labeled using the FOXP3/Transcription Factor Staining Buffer Set per the manufacturer’s protocol (Thermo Fisher Scientific, Waltham, MA), followed by labeling with antibodies obtained from Thermo Fisher, as listed below. To label T regulatory cells (Tregs, CD4^+^ FOXP3^+^), APC-labeled anti-CD4 (RM4-5) and FITC-labeled FOXP3 (FJK-16s) were used. To label Kupffer Cells [KCs, F4/80^+^ CD11b^lo^ LY6C^−^ (Alisi et al., 2017; Lynch et al., 2018)] and determine the M1 [CD11c^+^ CD206^-^) and M2 (CD11c^−^ CD206^+^ (Triantafyllou et al., 2021)] KC abundance, APC-labeled anti-CD11c (Bu15), PerCP-Cy5-labeled anti-CD11b (ICRF44), FITC-labeled anti-LY6C (RB6-8C5), PE-labeled anti-F4/80 (BM8), and Pacific Blue-labeled anti-CD206 (19.2) were used. Natural killer cells (NK1.1^+^) were identified with PE-labeled anti-NK1.1 (PK136). Finally, conventional cytotoxic T lymphocytes (TCRβ^+^ CD8a^+^) were identified with APC-labeled TCR beta (H57-597) and PerCP-Cy5-labeled anti-CD8a (53–6.7). Flow cytometry data was collected on a BD FACSCanto II Flow Cytometer and analyzed with FlowJo Software (v10.7, BD Biosciences, Franklin Lake, NJ). Gating strategy is summarized in Supplementary Figure S1. *n* = 3–4 mice per group were used.

### Western Blot Analysis

Liver tissue was homogenized by sonication in 20 mM Tris (pH 7.5), 2 mM EDTA, 10 mM EGTA, 1% Triton X-100, and protease/phosphatase inhibitors (Thermo Fisher Scientific). Insoluble material was removed by centrifugation at 10,000 × g for 10 min, and protein concentrations were measured (Bicinchoninic Acid Assay, Pierce Chemical Company, Rockford, IL). Samples (50 μg protein) were separated by SDS-PAGE, electroblotted onto nylon membranes (PVDF), and then probed with primary antibodies overnight at 4°C followed by a 1 h incubation with HRP-conjugated secondary antibodies (Thermo Fisher Scientific). Signals were visualized using Clarity Max™ Western ECL substrate and images were collected with the ChemiDoc™ imaging system and quantitated with Image Lab software, version 6.0.1 (Bio-Rad Laboratories, Hercules, CA). Anti-CYP2E1 antibodies were obtained from Abcam (Cambridge, MA, catalog number 28146), and anti-GAPDH antibodies from Cell Signaling Technology (Danvers, MA, catalog number 5147). *n* = 6 mice per group were chosen randomly of the 8–14 total mice for this analysis.

### Enzyme-Linked Immunosorbent Assay

Liver tissue was homogenized by sonication in 20 mM TRIS (pH 7.5), 2 mM EDTA, 10 mM EGTA, 1% Triton X-100, and 250 mM sucrose with HALT™ Protease and Phosphatase Inhibitor (Thermo Fisher Scientific), then centrifuged for 10 min at 10,000× g. Supernatants were collected, protein concentration was measured by BCA method (Thermo Fisher Scientific). CXCL2 was detected using the MesoScale Discovery Mouse MIP-2 V-Plex Kit as per manufacturer’s instructions (MesoScale Discovery, Rockville, MD), read on the MESO Sector S 600 Instrument, and analyzed with Discovery Workbench Software, v 4.0 (Meso Scale Discovery). PAI-1 was detected using the Mouse PAI-1 ELISA Kit according to the manufacturer’s instructions (Catalog No. EMSERPINE1, Thermo Fisher Scientific).

### Bone Marrow-Derived Macrophages

Bone marrow was flushed from the femurs and tibias of WT and *fat-1* mice, dissociated with a 21-gauge needle, and then passed through a 70 μm filter. Single cell suspensions were then plated in RPMI 1640 containing 10% FBS, penicillin, and streptomycin (Thermo Fisher Scientific). Cells were differentiated for 7 days into macrophages in the presence of 20% conditioned medium collected from cultured L929 cells (ATCC, Manassas, VA). Macrophage identity was verified by flow cytometry using PE-labeled anti-F4/80 (BM8) and PerCP-Cy5-labelled anti-Cd11b (ICRF44) antibodies (Thermo Fisher Scientific). Macrophages were then trypsinized and re-plated at 3.5 × 10^5^ cells/well in a 24-well plate for treatment. Cells were incubated in the presence of 100 mM EtOH for 24 h or 100 ng/ml LPS for 4 h before harvesting for RNA isolation and cDNA synthesis. Treatments were performed in triplicate. Each condition was performed in two independent experiments with similar results.

### Blood Alcohol Concentration Measurement

Blood alcohol concentration were determined in plasma using the EnzyChrom™ ethanol assay kit (San Jose, CA) according to the manufacturer’s instructions.

### PAI-1 Immunohistochemistry

Formalin-fixed, paraffin-embedded liver sections were deparaffinized and re-hydrated through graded EtOH solutions. Sections were then incubated in 20% goat serum and 0.2% Triton-X100 for 1 h at room temperature followed by an overnight incubation with a 1:100 dilution of anti-PAI-1 antibody (MA5-17171, Thermo Fisher Scientific). Sections were thoroughly washed with Tris-buffered saline + 0.1% Tween-20, incubated with HRP-conjugated anti-mouse antibodies and then developed with DAB (Dako/Agilent, Santa Clara, CA). Sections were de-hydrated and mounted under Cytoseal XYL mounting media (Thermo Fisher Scientific). Photomicrographs were taken with an Olympus BX43 microscope equipped with CellSens version 2.3 software (Olympus Life Science, Waltham, MA).

### Statistical Analyses

Differences among multiple groups were analyzed by unpaired one-way analysis of variance (ANOVA) followed by Sidak’s multiple comparison tests. Differences between two groups were analyzed by Student’s *t* test. All data are presented as the mean ± standard error of the mean. Analyses were performed using GraphPad Prism (GraphPad Software, version 9.0.1, San Diego, CA). *p* values < 0.05 were considered significant.

## Results

### *Fat-1* Mice Were Protected From Acute-On-Chronic-Ethanol-Induced Liver Injury

**FIGURE 1:**
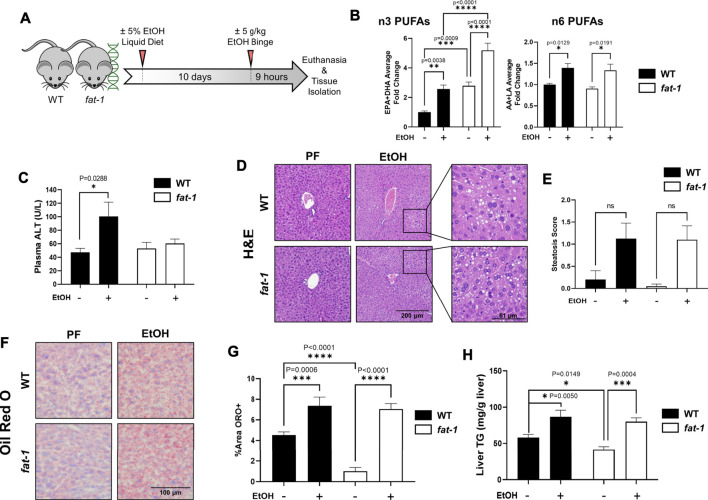
Characterization of liver pathology in WT and fat-1 mice in an acute-on-chronic mouse model of ALD. **(A)** Experimental design, **(B)** liver n3-PUFA (EPA and DHA) and n6-PUFA (AA and LA) levels, **(C)** plasma ALT, **(D)** H&E staining, **(E)** quantitation of liver steatosis, **(F)** Oil Red O staining, **(G)** quantitation of Oil Red O staining, and **(H)** total liver triglycerides.. All images are 200X magnification. *p < 0.05, **p < 0.01, ***p < 0.001, ****p < 0.0001, one-way ANOVA (comparisons not significant if unlabeled). WT PF (*n* = 14), *fat-1* PF (*n* = 9), WT EtOH (*n* = 8), *fat-1* EtOH (*n* = 10).

Male *fat-1* and WT littermates were placed on a diet containing EtOH for 10 days, followed by a bolus of EtOH delivered by oral gavage on the 11^th^ day to recapitulate human AH, an advanced stage of ALD (Bertola et al., 2013) (see Figure 1A for experimental design). Average food consumption and body weights for both WT and *fat-1* mice at the beginning and end of the experiment were similar, with both genotypes exhibiting a minor decrease in final body weights (Table 2). Both liver/body weight and epididymal fat/body weight ratios were also similar between WT and *fat-1* mice at the beginning and end of the experiment. There were no differences in blood alcohol concentration between WT and *fat-1* EtOH-treated mice (Table 2). Lastly, hepatic levels of n3-PUFAs were significantly higher in *fat-1* mice compared to WT mice both in PF and EtOH-fed groups (Figure 1B), whereas there were no differences in n6-PUFAs between genotypes. Interestingly, EtOH feeding resulted in an increase in both n6-and n3-PUFAs in WT and *fat-1* mice.

**TABLE 2:** Metabolic characteristics of WT and fat-1 mice in an acute-on-chronic model of ALD.

Characteristic	WT Pair-Fed	Fat-1 Pair-Fed	WT EtOH	Fat-1 EtOH
Food Consumption (g per day per mouse)	*	*	10.18 ± 0.64	9.19 ± 0.49
Weights				
Initial BW (g)	27.32 ± 0.86	26.83 ± 0.68	27.75 ± 0.43	27.39 ± 0.61
Final BW (g)	27.80 ± 0.84	26.67 ± 0.63	26.54 ± 0.37	26.80 ± 0.55
Body Weight Gain (%)	1.76 ± 0.04	−0.60 ± 0.03	^−^4.63 ± 0.02	^−^2.15 ± 0.03
Liver/BW Ratio (%)	3.50 ± 0.28	3.95 ± 0.14	4.00 ± 0.08	4.17 ± 0.11
Fat/BW Ratio (%)	0.14 ± 0.02	0.09 ± 0.01	0.12 ± 0.01	0.10 ± 0.01
Blood alcohol concentration (mM)	1.849 ± 0.20	2.001 ± 0.08	49.34 ± 17.45	40.52 ± 13.51

* PF mice consume the same amount of food as EtOH-fed mice, per genotype.

We observed a significant EtOH-induced increase in liver injury in WT mice, as demonstrated by elevation of plasma ALT levels, that was not evident in *fat-1* mice (Figure 1C). Analysis of H&E-stained liver sections revealed a similar overall morphology between WT and *fat-1* mice following EtOH treatment and demonstrated a similar level of microvesicular steatosis in both WT and *fat-1* mice (staining in Figure 1D and quantitation in Figure 1E). To better characterize the extent of hepatic steatosis, we performed Oil Red O staining for neutral lipids, which also demonstrated a similar degree of EtOH-induced steatosis in WT and *fat-1* mice (Figures 1F,G), further confirmed by a biochemical analysis of total liver TGs (Figure 1H). Interestingly, *fat-1* PF mice had significantly less steatosis than WT mice as measure by both Oil Red O and total TGs.

### Ethanol Administration Induced Similar Levels of Hepatic Oxidative Stress in Wild Type and *Fat-1* Mice

**FIGURE 2:**
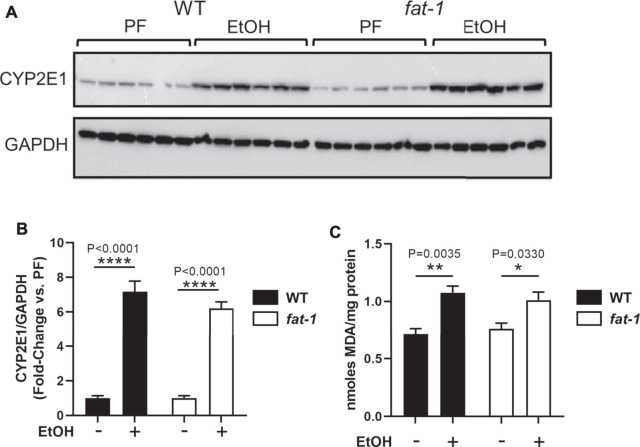
Hepatic expression of markers of oxidative stress. **(A,B)** Western blot and densitometric analysis for CYP2E1 and GAPDH expression. **(C)** TBARS assay to determine lipid peroxidation levels. *p < 0.05, **p < 0.01, ***p < 0.001, ****p < 0.0001, one-way ANOVA (comparisons not significant if unlabeled) *n* = 6 mice per group chosen randomly of the total 8–14.

We next determined if *fat-1* mice were protected from EtOH-associated oxidative stress, a key contributor to ALD pathogenesis (Leung and Nieto, 2013). EtOH consumption leads to oxidative stress, in part, through its metabolism by CYP2E1, a cytochrome P450 that produces reactive oxygen species as a byproduct of detoxification. EtOH also induces CYP2E1expression, thus leading to further reactive oxygen species production and oxidative stress. Western blotting analysis demonstrated that EtOH increased the expression of CYP2E1 by 6-7-fold in both WT and *fat-1* mice (Figures 2A,B). To determine resulting reactive oxygen species generation, we performed an assay for hepatic lipid peroxidation (TBARS) as an indirect a measure of oxidative stress and found a similar pattern as that for CYP2E1 expression. (Figure 2C). These data suggest that EtOH induction of oxidative stress was similar between WT and *fat-1* mice.

### Ethanol Treatment Caused Differential Effects on Markers of Hepatic Inflammation in *Fat-1* and Wild Type Mice

**FIGURE 3:**
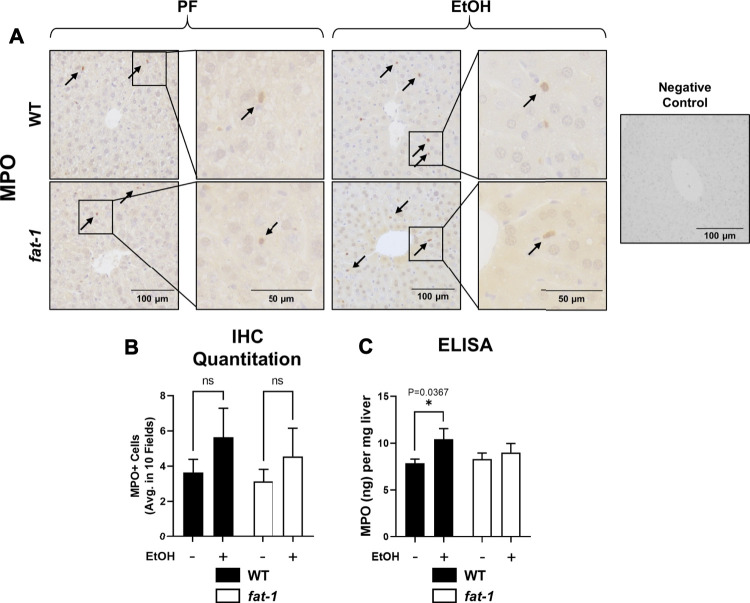
Hepatic neutrophil infiltration. **(A)** Immunohistochemistry staining for MPO expression. Arrows indicate MPO+ cells. Images are 200X, insets are 400X magnification. **(B)** Quantitation of number of MPO+ cells in representative digital microscope fields. **(C)** MPO levels as determined by ELISA, *p* < 0.05, one-way ANOVA (comparisons not significant if unlabeled). WT PF (*n* = 14), *fat-1* PF (*n* = 9), WT EtOH (*n* = 8), *fat-1* EtOH (*n* = 10).

**FIGURE 4:**
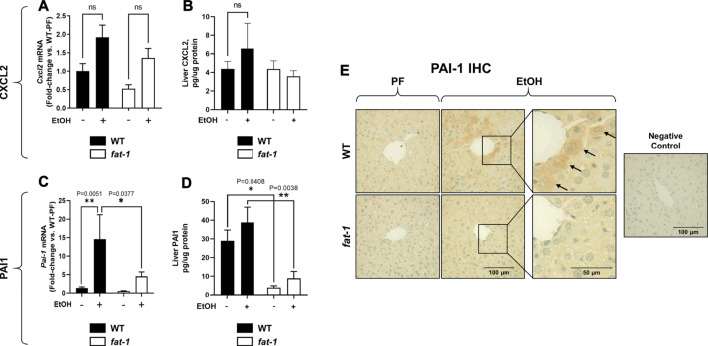
Hepatic Expression of Cxcl2 and Pai-1. **(A)** Expression of *Cxcl2* mRNA and **(B)** CXCL2 protein in liver. **(C)** Expression of *Pai-1* mRNA and **(D)** PAI-1 protein in liver. **(E)** Liver immunohistochemistry for PAI-1 protein, 400X magnification. Arrows indicate PAI-1+ hepatocytes. *, *p* < 0.05, **p < 0.01, one-way ANOVA (comparisons not significant if unlabeled). WT PF (*n* = 14), *fat-1* PF (*n* = 9), WT EtOH (*n* = 8), *fat-1* EtOH (*n* = 10).

Another pathological feature of ALD recapitulated by our acute-on-chronic EtOH treatment model is increased liver neutrophil infiltration (Bertola et al., 2013). To assay liver neutrophil accumulation, we measured liver myeloperoxidase (MPO) expression by both immunohistochemistry and ELISA (Figures 3A–C, respectively). While MPO immunohistochemistry showed no significant differences between groups, ELISA analysis of liver tissue lysates showed a significant increase in MPO levels in EtOH-fed vs PF WT mice which was not observed in *fat-1* mice. Neutrophils are recruited to the liver following injury by several chemokines, including CXCL2. Although the expression of whole-liver *Cxcl2* was increased (but not significantly) by EtOH in both WT and *fat-1* mice, there were no differences between the two genotypes (Figure 4A). CXCL2 protein in the liver was also modestly induced by EtOH, although again we observed no significant differences between genotypes (Figure 4B). Another mediator that can contribute to neutrophil chemoattraction is PAI-1, which is commonly elevated in both human ALD patients and in mouse models of ALD (Mukamal et al., 2001;Bergheim et al., 2006). PAI-1 is a pleiotropic acute phase response protein that has been classically associated with fibrin deposition but has been more recently identified to have a role in inflammation and/or neutrophil recruitment in some disease models (De Taeye et al., 2006;Praetner et al., 2018). In our study, the hepatic expression of *Pai-1* mRNA was significantly induced by EtOH in WT but not in *fat-1* mice (Figure 4C). At the protein level, EtOH had a limited effect on PAI-1 expression (Figure 4D). Importantly, *fat-1* mice expressed significantly less PAI-1 than WT mice (Figure 4D). Under certain conditions (*e.g.*, liver regeneration), hepatocytes can be a large source of PAI-1 (Thornton et al., 1994). Immunohistochemistry of liver sections revealed increased PAI-1 staining in hepatocytes of the pericentral region in WT EtOH vs WT PF mice, an induction which was not observed in *fat-1* EtOH-fed mice (Figure 4E). We also assayed the expression of additional markers of hepatic inflammation, namely IL-6 and TNF-α, but found no differences between PF or EtOH-treated *fat-1* and WT mice (data not shown).

**FIGURE 5:**
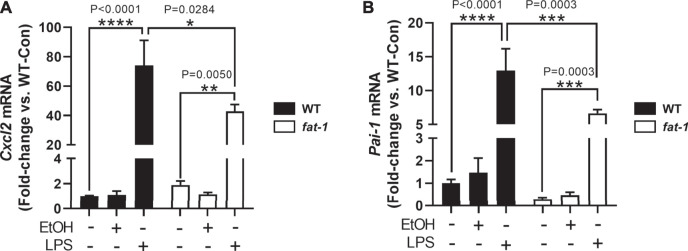
Expression of Cxcl2 and Pai-1 in BMDMs. **(A)** Expression of *Cxcl2* and **(B)** *Pai-1* in EtOH and LPS-treated BMDMs isolated from WT and *fat-1* mice. *p < 0.05, **p < 0.01, ***p < 0.001, ****p < 0.0001, one-way ANOVA (comparisons not significant if unlabeled). Experiment was performed twice with consistent results.

We next sought to determine other cell-specific contributions to the expression of neutrophil chemokines, *Cxcl2* and *Pai-1*, which are expressed by multiple cell types including macrophages (De Filippo et al., 2013; Fang et al., 2018). To determine the expression of *Cxcl2* and *Pai-1* expression in macrophages, we isolated bone marrow cells from WT and *fat-1* mice and differentiated them into macrophages (BMDMs). Notably, baseline expression of *Pai-1* was lower, albeit not significantly, in *fat-1*-derived BMDMs than in WT-derived BMDMs (Figure 5B). Incubation with EtOH had no significant effect on either *Cxcl2* (Figure 5A) or *Pai-1* (Figure 5B) expression in both *fat-1* and WT BMDMs. However, LPS stimulation led to a large increase in both *Cxcl2* and *Pai-1* expression in WT and *fat-1*-derived BMDMs, but with significantly less induction in *fat-1* BMDMs.

### *Fat-1* Genotype Favorably Altered Hepatic Immune Cell Phenotype and Abundance in Response to Ethanol Challenge

**FIGURE 6:**
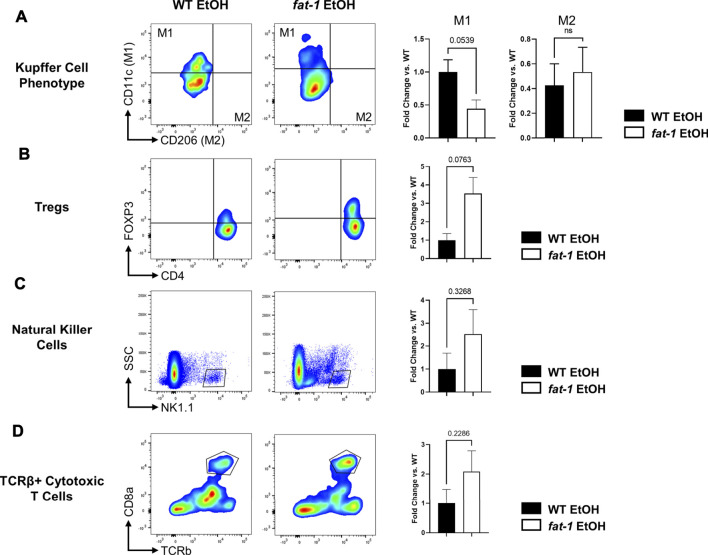
Flow cytometry analysis of hepatic immune cells following EtOH feeding. Abundance of **(A)** M1 and M2 KCs, **(B)** T regulatory cells, **(C)** Natural killer cells, and **(D)** TCRβ+ killer cells. *, *p* < 0.05, two-tailed Student’s *t* test. Results are an average of *n* = 3–4 mice per group.

To investigate the effects of n3-PUFA enrichment on liver immunity, we analyzed several liver immune cell populations by flow cytometry. First, we found that *fat-1* EtOH-treated mice had decreased populations of M1 KCs relative to WT EtOH-treated mice with no change in M2 KCs, indicating a shift away from the pro-inflammatory M1 KC phenotype (Figure 6A). We also noted an increase in liver anti-inflammatory Tregs in *fat-1* EtOH-treated mice *vs* WT EtOH-treated mice (Figure 6B), a cell type which is critical for maintaining immune homeostasis and self-tolerance, as well as blocking pro-inflammatory signals (Corthay, 2009). There were also trending increases in additional liver immune cells including natural killer cells as well as conventional cytotoxic T cells, indicating multiple effects of n3-PUFA enrichment on liver innate and adaptive immunity (Figures 6C,D).

## Discussion

In the current study, we interrogated the effects of endogenous n3-PUFA enrichment in an animal model that mimics AH, a severe stage of human ALD. The significance of this study is based on the evidence that patients with severe alcohol-associated liver disease (including AH) have decreased levels of critical n3-PUFAs such as EPA and DHA (or their metabolites) compared to healthy controls or alcohol-dependent individuals without major liver disease (Johnson et al., 1985; Gao et al., 2019). Here, we demonstrated that transgenic *fat-1* mice, a strain with significantly elevated n3-PUFA levels due to endogenous conversion of n6-PUFAs to n3-PUFAs, are protected against experimental ALD. Interestingly, unlike in human studies, acute-on-chronic EtOH administration in our mouse model resulted in an increase in n3-PUFAs in both genotypes, although *fat-1* mice still had significantly higher n3-PUFAs compared to WT mice. Along with n3-PUFAs, EtOH also induced n6-PUFAs; the exact molecular mechanism responsible for this increase remains to be investigated. A similar phenomenon of elevated n3-PUFA levels was previously observed in the livers of mice chronically fed EtOH (Hardesty et al., 2021; Warner et al., 2021), and in the plasma of EtOH-fed rats (Guiraud et al., 2008); however, no change in n3-PUFAs with acute-on-chronic EtOH feeding has also been reported (Wang et al., 2017). This discrepancy may be due to differences in the animal models (e.g., differences in the diet composition, the amount and duration of EtOH administration, etc.), or differences in the gut microbiota, a known player in fatty acid (Kindt et al., 2018) and EtOH metabolism (Zhu et al., 2013) in the intestine as well as susceptibility to ALD (Llopis et al., 2016).

The mechanism(s) affording protection against EtOH-associated liver injury in *fat-1* mice in our study appear not to be through inhibiting hepatic steatosis, reducing hepatic oxidative stress, or altering EtOH metabolism. The primary effects that we found pertained instead to hepatic immune cells, including decreased neutrophil infiltration. During initial phases of the inflammatory response, neutrophils play a beneficial role by producing pro-inflammatory cytokines and attacking microorganisms through several mechanisms including phagocytosis, degranulation, and respiratory burst (Nemeth et al., 2020). However, their persistence can ultimately damage healthy liver tissue (Wilgus et al., 2013; Wang, 2018). Therefore, proper clearance of neutrophils through efferocytosis by macrophages or elimination of chemotactic signals is essential to regulate neutrophil accumulation in the liver following a toxic insult. However, EtOH consumption leads to decreased neutrophil clearance, prolonged expression of neutrophil chemotactic signals, as well as dysregulated neutrophil function in both human ALD and experimental mouse models (Bautista, 2002; Das et al., 2017; Bukong et al., 2018).

While there were no significant global differences in the hepatic expression of the canonical neutrophil chemoattractant CXCL2, we found a significant reduction in the expression of *Pai-1* mRNA in *fat-1* EtOH-fed mice compared to WT EtOH-fed littermates. Although PAI-1 protein levels did not entirely follow expression of *Pai-1* mRNA, it was clear that *fat-1* mice express far less *Pai-1* than their WT counterparts. The most thoroughly characterized function of PAI-1 is in regulating fibrin formation (Morrow et al., 2021), giving it a central role in liver fibrosis (Wang et al., 2007), which is a hallmark of later stages of ALD. However, PAI-1 can also serve as a chemoattractant for and apoptosis inhibitor of neutrophils (Marshall et al., 2003; Zmijewski et al., 2011). Neutrophil apoptosis is a key process for recognition and eventual efferocytosis by macrophages. Therefore, we propose that one of the key mechanisms by which *fat-1* mice and n3-PUFAs can ameliorate liver injury is by attenuating expression of *Pai-1*. Decreased *Pai-1* expression in *fat-1* mice may not only decrease hepatic neutrophil infiltration but could also enhance the clearance of these cells in the liver, in turn leading to a decreased risk of damage to liver tissue. Of note, we identified cell-specific effects (specifically on BMDMs) of n3-PUFA enrichment, where BMDMs derived from *fat-1* mice had decreased expression of *Cxcl2* and *Pai-1* relative to BMDMs obtained from WT mice, suggesting a role for macrophages in the protective effects of n3-PUFAs in this context. The favorable effects of *Pai-1* reduction on EtOH-induced liver injury in our study are consistent with a previous report from the Dr. Arteel group demonstrating that genetic deletion of *Pai-1* prevented development of liver injury and inflammation due to chronic EtOH administration (Bergheim et al., 2006), suggesting that PAI-1 is an important pathogenic inflammatory mediator in ALD.

Importantly, our previous work showed a decreased *Pai-1* expression in *fat-1* mice in another animal model (chronic EtOH feeding) which produces early-stage features of ALD (Hardesty et al., 2021), suggesting that endogenous n3-PUFA enrichment is able to down-regulate *Pai-1* in multiple stages of ALD severity. The exact mechanisms by which n3-PUFA enrichment decreased *Pai-1* expression remains to be determined in future studies, but this downregulation may be due to a decreased n6/n3-PUFA ratio, as linoleic acid (an n6-PUFA) has been shown to induce PAI-1 expression in HepG2 cells (Banfi et al., 1997). In addition, *fat-1* mice have increased levels of specialized pro-resolving mediators such as resolvins (Hudert et al., 2006), a group of molecules which can decrease *PAI-1* expression in human macrophages (Gilligan et al., 2019).

Another important observation in our study was that *fat-*1 EtOH-fed mice, compared to WT *EtOH*-fed mice, had several favorable changes in hepatic immune cell populations which may also contribute to attenuated liver injury in *fat-1* mice. First, *fat-1* mice had decreased abundance of M1 KCs, indicating a shift away from a pro-inflammatory macrophage phenotype. KCs are liver-resident macrophages which play a central role as mediators of liver injury and repair, including in ALD (Kamimura and Tsukamoto, 1995). The activated M1 phenotype is associated with increased pro-inflammatory signaling whereas the M2 phenotype is associated with increased anti-inflammatory signaling, contributing to resolution of inflammation and return to liver tissue homeostasis (Dixon et al., 2013). Further, we observed increased Treg abundance in *fat-1* but not WT mice in response to EtOH treatment. Tregs are increasingly understood to be a beneficial cell population which blunt inflammation in liver disease in both mice and humans [e.g., NAFLD (Ma et al., 2007; Van Herck et al., 2019)], and it has been shown that Tregs are depleted in human AH patients (Almeida et al., 2013). Importantly, Tregs have been shown to inhibit neutrophil accumulation (Richards et al., 2010), and conversely, loss of Tregs reduces neutrophil apoptosis and increases MPO activity (Okeke et al., 2017). Other studies demonstrate that Tregs inhibit neutrophil function and increase their apoptosis (Lewkowicz et al., 2006), altogether suggesting that Tregs may contribute to decreased neutrophil accumulation in our *fat-1* mice in addition to decreased *Pai-1*. Lastly, NK cell abundance was slightly increased in *fat-1* mice; NK cells are key members of innate immunity which contribute to protection against viral hepatitis, liver fibrosis, and liver tumorigenesis (Tian et al., 2013). A previous study supports a positive correlation between NK infiltration and NK activation, indicating NK activation may be increased in *fat-1* mice (Li et al., 2020).

**FIGURE 7:**
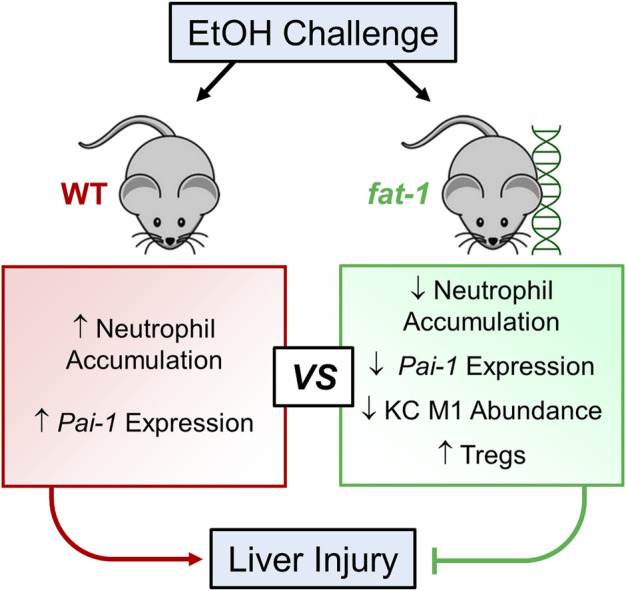
Schematic representation of the beneficial effects of n3-PUFA enrichment on liver injury in acute-on-chronic EtOH-induced experimental ALD.

Taken together, our results demonstrated that n3-PUFA enrichment in *fat-1* mice attenuated EtOH-induced liver injury, and suggest that this effect is mediated, in part, via reduction of neutrophil accumulation, a decrease in hepatic *Pai-1* expression, decreased M1 macrophage abundance, and increased liver Treg abundance (Figure 7). Our results contribute to a growing body of evidence that dietary enrichment in n3-PUFAs represents a promising nutritional adjuvant therapy for ALD.

## Data Availability

The raw data supporting the conclusions of this article will be made available by the authors, without undue reservation.

## References

[B1] Alisi A. Carpino G. Oliveira F. L. Panera N. Nobili V. Gaudio E. (2017). The Role of Tissue Macrophage-Mediated Inflammation on NAFLD Pathogenesis and its Clinical Implications. Mediators Inflamm. 2017, 8162421. 10.1155/2017/8162421 28115795 PMC5237469

[B2] Almeida J. Polvorosa M. A. Gonzalez-Quintela A. Marcos M. Pastor I. Hernandez Cerceño M. L. (2013). Decreased Peripheral Blood CD4+/CD25+ Regulatory T Cells in Patients with Alcoholic Hepatitis. Alcohol. Clin. Exp. Res. 37, 1361–1369. 10.1111/acer.12095 23550693

[B3] Banfi C. Risé P. Mussoni L. Galli C. Tremoli E. (1997). Linoleic Acid Enhances the Secretion of Plasminogen Activator Inhibitor Type 1 by HepG2 Cells. J. Lipid Res. 38, 860–869. 10.1016/s0022-2275(20)37211-4 9186904

[B4] Bautista A. P. (2002). Neutrophilic Infiltration in Alcoholic Hepatitis. Alcohol 27, 17–21. 10.1016/s0741-8329(02)00206-9 12062632

[B5] Bedossa P. Consortium F. P. (2014). Utility and Appropriateness of the Fatty Liver Inhibition of Progression (FLIP) Algorithm and Steatosis, Activity, and Fibrosis (SAF) Score in the Evaluation of Biopsies of Nonalcoholic Fatty Liver Disease. Hepatology 60, 565–575. 10.1002/hep.27173 24753132

[B6] Bergheim I. Guo L. Davis M. A. Lambert J. C. Beier J. I. Duveau I. (2006). Metformin Prevents Alcohol-Induced Liver Injury in the Mouse: Critical Role of Plasminogen Activator Inhibitor-1. Gastroenterology 130, 2099–2112. 10.1053/j.gastro.2006.03.020 16762632 PMC2648856

[B7] Bertola A. Mathews S. Ki S. H. Wang H. Gao B. (2013). Mouse Model of Chronic and Binge Ethanol Feeding (The NIAAA Model). Nat. Protoc. 8, 627–637. 10.1038/nprot.2013.032 23449255 PMC3788579

[B8] Bukong T. N. Cho Y. Iracheta-Vellve A. Saha B. Lowe P. Adejumo A. (2018). Abnormal Neutrophil Traps and Impaired Efferocytosis Contribute to Liver Injury and Sepsis Severity after Binge Alcohol Use. J. Hepatol. 69, 1145–1154. 10.1016/j.jhep.2018.07.005 30030149 PMC6310218

[B9] Chiang D. J. Mccullough A. J. (2014). The Impact of Obesity and Metabolic Syndrome on Alcoholic Liver Disease. Clin. Liver Dis. 18, 157–163. 10.1016/j.cld.2013.09.006 24274871 PMC6130318

[B10] Chu S. Sun R. Gu X. Chen L. Liu M. Guo H. (2020). Inhibition of Sphingosine-1-Phosphate-Induced Th17 Cells Ameliorates Alcoholic Steatohepatitis in Mice. Hepatology 73 (3), 952–967. 10.1002/hep.31321 PMC8009334 32418220

[B11] Corthay A. (2009). How Do Regulatory T Cells Work? Scand. J. Immunol. 70, 326–336. 10.1111/j.1365-3083.2009.02308.x 19751267 PMC2784904

[B12] Crabb D. W. Im G. Y. Szabo G. Mellinger J. L. Lucey M. R. (2020). Diagnosis and Treatment of Alcohol-Associated Liver Diseases: 2019 Practice Guidance from the American Association for the Study of Liver Diseases. Hepatology 71, 306–333. 10.1002/hep.30866 31314133

[B13] Dalli J. Colas R. A. Serhan C. N. (2013). Novel N-3 Immunoresolvents: Structures and Actions. Sci. Rep. 3, 1940. 10.1038/srep01940 23736886 PMC3672887

[B14] Das S. Maras J. S. Hussain M. S. Sharma S. David P. Sukriti S. (2017). Hyperoxidized Albumin Modulates Neutrophils to Induce Oxidative Stress and Inflammation in Severe Alcoholic Hepatitis. Hepatology 65, 631–646. 10.1002/hep.28897 27775820

[B15] De Filippo K. Dudeck A. Hasenberg M. Nye E. Van Rooijen N. Hartmann K. (2013). Mast Cell and Macrophage Chemokines CXCL1/CXCL2 Control the Early Stage of Neutrophil Recruitment during Tissue Inflammation. Blood 121, 4930–4937. 10.1182/blood-2013-02-486217 23645836

[B16] De Taeye B. M. Novitskaya T. Gleaves L. Covington J. W. Vaughan D. E. (2006). Bone Marrow Plasminogen Activator Inhibitor-1 Influences the Development of Obesity. J. Biol. Chem. 281, 32796–32805. 10.1074/jbc.M606214200 16931518

[B17] Dixon L. J. Barnes M. Tang H. Pritchard M. T. Nagy L. E. (2013). Kupffer Cells in the Liver. Compr. Physiol. 3, 785–797. 10.1002/cphy.c120026 23720329 PMC4748178

[B18] Fang W. F. Chen Y. M. Lin C. Y. Huang H. L. Yeh H. Chang Y. T. (2018). Histone Deacetylase 2 (HDAC2) Attenuates Lipopolysaccharide (LPS)-induced Inflammation by Regulating PAI-1 Expression. J. Inflamm. (Lond) 15, 3. 10.1186/s12950-018-0179-6 29344006 PMC5763578

[B19] Fung P. Pyrsopoulos N. (2017). Emerging Concepts in Alcoholic Hepatitis. World J. Hepatol. 9, 567–585. 10.4254/wjh.v9.i12.567 28515843 PMC5411952

[B20] Gao B. Lang S. Duan Y. Wang Y. Shawcross D. L. Louvet A. (2019). Serum and Fecal Oxylipins in Patients with Alcohol-Related Liver Disease. Dig. Dis. Sci. 64, 1878–1892. 10.1007/s10620-019-05638-y 31076986 PMC6588282

[B21] Gilligan M. M. Gartung A. Sulciner M. L. Norris P. C. Sukhatme V. P. Bielenberg D. R. (2019). Aspirin-triggered Proresolving Mediators Stimulate Resolution in Cancer. Proc. Natl. Acad. Sci. U S A. 116, 6292–6297. 10.1073/pnas.1804000116 30862734 PMC6442621

[B22] Guiraud A. De Lorgeril M. Zeghichi S. Laporte F. Salen P. Saks V. (2008). Interactions of Ethanol Drinking with N-3 Fatty Acids in Rats: Potential Consequences for the Cardiovascular System. Br. J. Nutr. 100, 1237–1244. 10.1017/S0007114508981472 18445308

[B23] Hardesty J. E. Warner J. B. Song Y. L. Rouchka E. C. Mcclain C. J. Warner D. R. (2021). Ileum Gene Expression in Response to Acute Systemic Inflammation in Mice Chronically Fed Ethanol: Beneficial Effects of Elevated Tissue N-3 PUFAs. Int. J. Mol. Sci. 22 (4), 1582. 10.3390/ijms22041582 33557303 PMC7914826

[B24] Huang W. Wang B. Li X. Kang J. X. (2015). Endogenously Elevated N-3 Polyunsaturated Fatty Acids Alleviate Acute Ethanol-Induced Liver Steatosis. Biofactors 41, 453–462. 10.1002/biof.1246 26637972

[B25] Hudert C. A. Weylandt K. H. Lu Y. Wang J. Hong S. Dignass A. (2006). Transgenic Mice Rich in Endogenous omega-3 Fatty Acids Are Protected from Colitis. Proc. Natl. Acad. Sci. U S A. 103, 11276–11281. 10.1073/pnas.0601280103 16847262 PMC1544078

[B26] Jaeschke H. (2002). Neutrophil-mediated Tissue Injury in Alcoholic Hepatitis. Alcohol 27, 23–27. 10.1016/s0741-8329(02)00200-8 12062633

[B27] Johnson S. B. Gordon E. Mcclain C. Low G. Holman R. T. (1985). Abnormal Polyunsaturated Fatty Acid Patterns of Serum Lipids in Alcoholism and Cirrhosis: Arachidonic Acid Deficiency in Cirrhosis. Proc. Natl. Acad. Sci. U S A. 82, 1815–1818. 10.1073/pnas.82.6.1815 3920655 PMC397363

[B28] Kamimura S. Tsukamoto H. (1995). Cytokine Gene Expression by Kupffer Cells in Experimental Alcoholic Liver Disease. Hepatology 22, 1304–1309. 10.1002/hep.1840220441 7557885

[B29] Kang J. X. Wang J. Wu L. Kang Z. B. (2004). Transgenic Mice: Fat-1 Mice Convert N-6 to N-3 Fatty Acids. Nature 427, 504. 10.1038/427504a 14765186

[B30] Kindt A. Liebisch G. Clavel T. Haller D. Hörmannsperger G. Yoon H. (2018). The Gut Microbiota Promotes Hepatic Fatty Acid Desaturation and Elongation in Mice. Nat. Commun. 9, 3760. 10.1038/s41467-018-05767-4 30218046 PMC6138742

[B31] Kirpich I. A. Feng W. Wang Y. Liu Y. Barker D. F. Barve S. S. (2012). The Type of Dietary Fat Modulates Intestinal Tight junction Integrity, Gut Permeability, and Hepatic Toll-like Receptor Expression in a Mouse Model of Alcoholic Liver Disease. Alcohol. Clin. Exp. Res. 36, 835–846. 10.1111/j.1530-0277.2011.01673.x 22150547 PMC3319492

[B32] Kirpich I. A. Miller M. E. Cave M. C. Joshi-Barve S. Mcclain C. J. (2016). Alcoholic Liver Disease: Update on the Role of Dietary Fat. Biomolecules 6, 1. 10.3390/biom6010001 26751488 PMC4808795

[B33] Leung T. M. Nieto N. (2013). CYP2E1 and Oxidant Stress in Alcoholic and Non-alcoholic Fatty Liver Disease. J. Hepatol. 58, 395–398. 10.1016/j.jhep.2012.08.018 22940046

[B34] Lewkowicz P. Lewkowicz N. Sasiak A. Tchórzewski H. (2006). Lipopolysaccharide-activated CD4+CD25+ T Regulatory Cells Inhibit Neutrophil Function and Promote Their Apoptosis and Death. J. Immunol. 177, 7155–7163. 10.4049/jimmunol.177.10.7155 17082633

[B35] Li B. Jiang Y. Li G. Fisher G. A. Jr. Li R. (2020). Natural Killer Cell and Stroma Abundance Are Independently Prognostic and Predict Gastric Cancer Chemotherapy Benefit. JCI Insight 5. 10.1172/jci.insight.136570 PMC7253031 32229725

[B36] Livak K. J. Schmittgen T. D. (2001). Analysis of Relative Gene Expression Data Using Real-Time Quantitative PCR and the 2(-Delta Delta C(T)) Method. Methods 25, 402–408. 10.1006/meth.2001.1262 11846609

[B37] Llopis M. Cassard A. M. Wrzosek L. Boschat L. Bruneau A. Ferrere G. (2016). Intestinal Microbiota Contributes to Individual Susceptibility to Alcoholic Liver Disease. Gut 65, 830–839. 10.1136/gutjnl-2015-310585 26642859

[B38] Lynch R. W. Hawley C. A. Pellicoro A. Bain C. C. Iredale J. P. Jenkins S. J. (2018). An Efficient Method to Isolate Kupffer Cells Eliminating Endothelial Cell Contamination and Selective Bias. J. Leukoc. Biol. 104, 579–586. 10.1002/JLB.1TA0517-169R 29607532 PMC6175317

[B39] Ma X. Hua J. Mohamood A. R. Hamad A. R. Ravi R. Li Z. (2007). A High-Fat Diet and Regulatory T Cells Influence Susceptibility to Endotoxin-Induced Liver Injury. Hepatology 46, 1519–1529. 10.1002/hep.21823 17661402

[B40] Marshall L. J. Ramdin L. S. Brooks T. DPhil P. C. Shute J. K. (2003). Plasminogen Activator Inhibitor-1 Supports IL-8-mediated Neutrophil Transendothelial Migration by Inhibition of the Constitutive Shedding of Endothelial IL-8/heparan Sulfate/syndecan-1 Complexes. J. Immunol. 171, 2057–2065. 10.4049/jimmunol.171.4.2057 12902511

[B41] Mathurin P. Bataller R. (2015). Trends in the Management and burden of Alcoholic Liver Disease. J. Hepatol. 62, S38–S46. 10.1016/j.jhep.2015.03.006 25920088 PMC5013530

[B42] Mcclain C. J. Barve S. S. Barve A. Marsano L. (2011). Alcoholic Liver Disease and Malnutrition. Alcohol. Clin. Exp. Res. 35, 815–820. 10.1111/j.1530-0277.2010.01405.x 21284673 PMC3771636

[B43] Meroni M. Longo M. Rametta R. Dongiovanni P. (2018). Genetic and Epigenetic Modifiers of Alcoholic Liver Disease. Int. J. Mol. Sci. 19. 10.3390/ijms19123857 PMC6320903 30513996

[B44] Morrow G. B. Whyte C. S. Mutch N. J. (2021). A Serpin with a Finger in Many PAIs: PAI-1's Central Function in Thromboinflammation and Cardiovascular Disease. Front. Cardiovasc. Med. 8, 653655. 10.3389/fcvm.2021.653655 33937363 PMC8085275

[B45] Mukamal K. J. Jadhav P. P. D'agostino R. B. Massaro J. M. Mittleman M. A. Lipinska I. (2001). Alcohol Consumption and Hemostatic Factors: Analysis of the Framingham Offspring Cohort. Circulation 104, 1367–1373. 10.1161/hc3701.096067 11560851

[B46] Németh T. Sperandio M. Mócsai A. (2020). Neutrophils as Emerging Therapeutic Targets. Nat. Rev. Drug Discov. 19, 253–275. 10.1038/s41573-019-0054-z 31969717

[B47] Okeke E. B. Mou Z. Onyilagha N. Jia P. Gounni A. S. Uzonna J. E. (2017). Deficiency of Phosphatidylinositol 3-Kinase δ Signaling Leads to Diminished Numbers of Regulatory T Cells and Increased Neutrophil Activity Resulting in Mortality Due to Endotoxic Shock. J. Immunol. 199, 1086–1095. 10.4049/jimmunol.1600954 28659355

[B48] Patterson E. Wall R. Fitzgerald G. F. Ross R. P. Stanton C. (2012). Health Implications of High Dietary omega-6 Polyunsaturated Fatty Acids. J. Nutr. Metab. 2012, 539426. 10.1155/2012/539426 22570770 PMC3335257

[B49] Praetner M. Zuchtriegel G. Holzer M. Uhl B. Schaubächer J. Mittmann L. (2018). Plasminogen Activator Inhibitor-1 Promotes Neutrophil Infiltration and Tissue Injury on Ischemia-Reperfusion. Arterioscler Thromb. Vasc. Biol. 38, 829–842. 10.1161/ATVBAHA.117.309760 29371242

[B50] Richards H. Williams A. Jones E. Hindley J. Godkin A. Simon A. K. (2010). Novel Role of Regulatory T Cells in Limiting Early Neutrophil Responses in Skin. Immunology 131, 583–592. 10.1111/j.1365-2567.2010.03333.x 20722759 PMC2999808

[B51] Seitz H. K. Bataller R. Cortez-Pinto H. Gao B. Gual A. Lackner C. (2018). Alcoholic Liver Disease. Nat. Rev. Dis. Primers 4, 16. 10.1038/s41572-018-0014-7 30115921

[B52] Thornton A. J. Bruzdzinski C. J. Raper S. E. Gelehrter T. D. (1994). Plasminogen Activator Inhibitor-1 Is an Immediate Early Response Gene in Regenerating Rat Liver. Cancer Res. 54, 1337–1343. 8118825

[B53] Tian Z. Chen Y. Gao B. (2013). Natural Killer Cells in Liver Disease. Hepatology 57, 1654–1662. 10.1002/hep.26115 23111952 PMC3573257

[B54] Triantafyllou E. Gudd C. L. Mawhin M. A. Husbyn H. C. Trovato F. M. Siggins M. K. (2021). PD-1 Blockade Improves Kupffer Cell Bacterial Clearance in Acute Liver Injury. J. Clin. Invest. 131, e140196. 10.1172/JCI140196 PMC7880414 33320839

[B55] Tull S. P. Yates C. M. Maskrey B. H. O'donnell V. B. Madden J. Grimble R. F. (2009). Omega-3 Fatty Acids and Inflammation: Novel Interactions Reveal a New Step in Neutrophil Recruitment. Plos Biol. 7, e1000177. 10.1371/journal.pbio.1000177 19707265 PMC2718617

[B56] Van Herck M. A. Weyler J. Kwanten W. J. Dirinck E. L. De Winter B. Y. Francque S. M. (2019). The Differential Roles of T Cells in Non-alcoholic Fatty Liver Disease and Obesity. Front. Immunol. 10, 82. 10.3389/fimmu.2019.00082 30787925 PMC6372559

[B57] Wang H. Zhang Y. Heuckeroth R. O. (2007). PAI-1 Deficiency Reduces Liver Fibrosis after Bile Duct Ligation in Mice through Activation of tPA. FEBS Lett. 581, 3098–3104. 10.1016/j.febslet.2007.05.049 17561000

[B58] Wang J. (2018). Neutrophils in Tissue Injury and Repair. Cell Tissue Res 371, 531–539. 10.1007/s00441-017-2785-7 29383445 PMC5820392

[B59] Wang M. Zhang X. Ma L. J. Feng R. B. Yan C. Su H. (2017). Omega-3 Polyunsaturated Fatty Acids Ameliorate Ethanol-Induced Adipose Hyperlipolysis: A Mechanism for Hepatoprotective Effect against Alcoholic Liver Disease. Biochim. Biophys. Acta Mol. Basis Dis. 1863, 3190–3201. 10.1016/j.bbadis.2017.08.026 28847514

[B60] Warner D. R. Liu H. Ghosh Dastidar S. Warner J. B. Prodhan M. A. I. Yin X. (2018). Ethanol and Unsaturated Dietary Fat Induce Unique Patterns of Hepatic ω-6 and ω-3 PUFA Oxylipins in a Mouse Model of Alcoholic Liver Disease. PLoS One 13, e0204119. 10.1371/journal.pone.0204119 30256818 PMC6157879

[B61] Warner D. R. Liu H. Miller M. E. Ramsden C. E. Gao B. Feldstein A. E. (2017). Dietary Linoleic Acid and its Oxidized Metabolites Exacerbate Liver Injury Caused by Ethanol via Induction of Hepatic Proinflammatory Response in Mice. Am. J. Pathol. 187, 2232–2245. 10.1016/j.ajpath.2017.06.008 28923202 PMC5808136

[B62] Warner D. R. Warner J. B. Hardesty J. E. Song Y. L. Chen C. Y. Chen Z. (2021). Beneficial Effects of an Endogenous Enrichment in N3-PUFAs on Wnt Signaling Are Associated with Attenuation of Alcohol-Mediated Liver Disease in Mice. FASEB J. 35, e21377. 10.1096/fj.202001202R 33481293 PMC8243414

[B63] Warner D. R. Warner J. B. Hardesty J. E. Song Y. L. King T. N. Kang J. X. (2019). Decreased ω-6:ω-3 PUFA Ratio Attenuates Ethanol-Induced Alterations in Intestinal Homeostasis, Microbiota, and Liver Injury. J. Lipid Res. 60, 2034–2049. 10.1194/jlr.RA119000200 31586017 PMC6889711

[B64] Wilgus T. A. Roy S. Mcdaniel J. C. (2013). Neutrophils and Wound Repair: Positive Actions and Negative Reactions. Adv. Wound Care (New Rochelle) 2, 379–388. 10.1089/wound.2012.0383 24527354 PMC3763227

[B65] Yuan F. He L. Feng W. Mcclain C. J. Zhang X. (2020). High-Throughput Profiling of Long Chain Fatty Acids and Oxylipins by LC–MS. Curr. Trends Mass Spectrom. 18, 28–34.

[B66] Zhu L. Baker S. S. Gill C. Liu W. Alkhouri R. Baker R. D. (2013). Characterization of Gut Microbiomes in Nonalcoholic Steatohepatitis (NASH) Patients: a Connection between Endogenous Alcohol and NASH. Hepatology 57, 601–609. 10.1002/hep.26093 23055155

[B67] Zirnheld K. H. Warner D. R. Warner J. B. Hardesty J. E. Mcclain C. J. Kirpich I. A. (2019). Dietary Fatty Acids and Bioactive Fatty Acid Metabolites in Alcoholic Liver Disease. Liver Res. 3, 206–217. 10.1016/j.livres.2019.10.001

[B68] Zmijewski J. W. Bae H. B. Deshane J. S. Peterson C. B. Chaplin D. D. Abraham E. (2011). Inhibition of Neutrophil Apoptosis by PAI-1. Am. J. Physiol. Lung Cel Mol Physiol 301, L247–L254. 10.1152/ajplung.00075.2011 PMC3154633 21622848

