# Supplementary material for: Fat-1 Transgenic Mice With Augmented n3-Polyunsaturated Fatty Acids Are Protected From Liver Injury Caused by Acute-On-Chronic Ethanol Administration
Source: Front Pharmacol. 2021 Aug 31;12:711590. doi: 10.3389/fphar.2021.711590 (PMC8438569; doi:10.3389/fphar.2021.711590)
Supplement: Supplementary file 1 [file Presentation1.PPTX]

## Slide 1
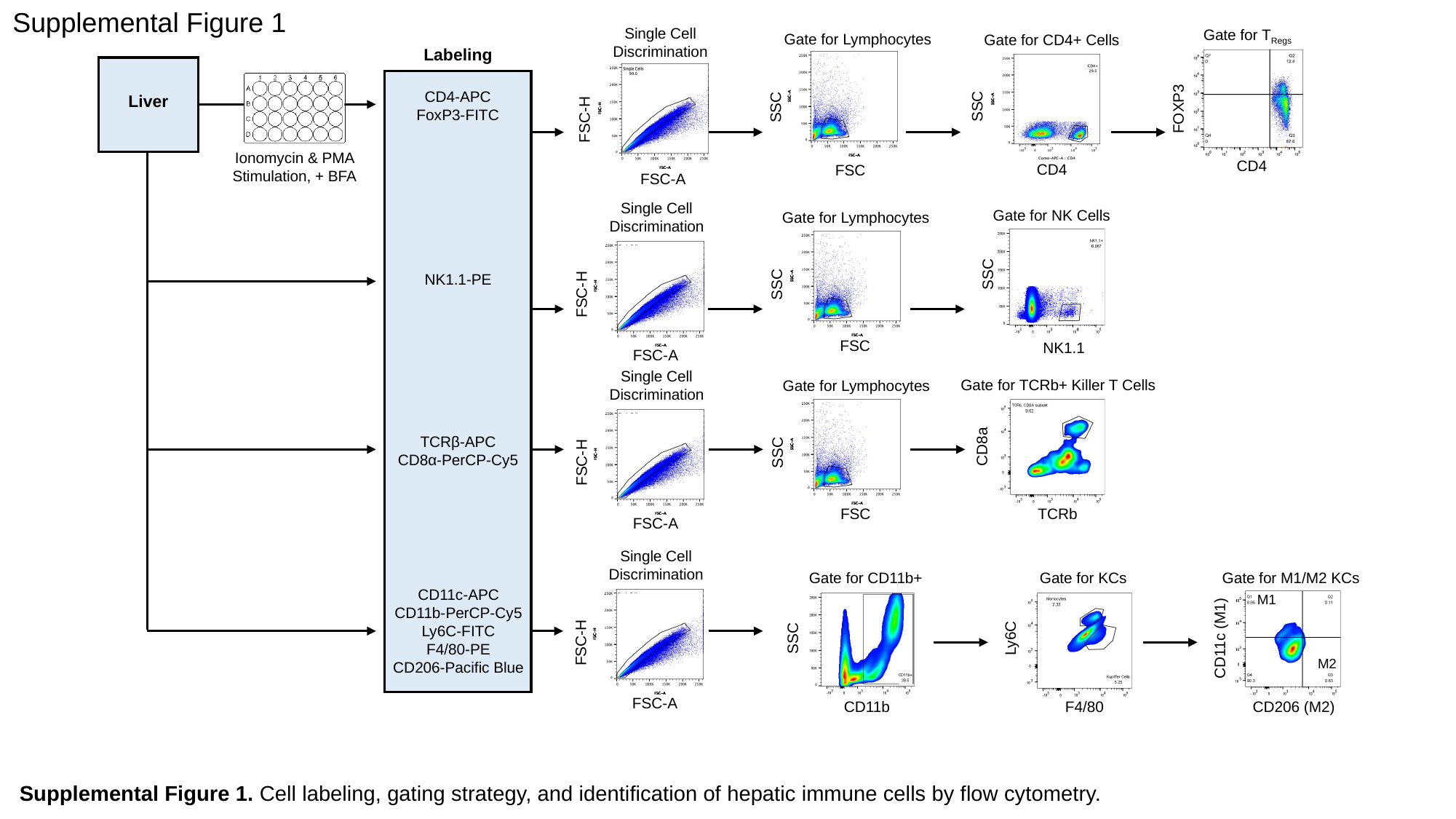

Supplemental Figure 1
Single Cell Discrimination
FSC-H
FSC-A
Gate for TRegs
FOXP3
CD4
Gate for Lymphocytes
SSC
FSC
Gate for CD4+ Cells
SSC
CD4
Labeling
Liver
Ionomycin & PMA Stimulation, + BFA
Single Cell Discrimination
FSC-H
FSC-A
Gate for NK Cells
SSC
NK1.1
Gate for Lymphocytes
SSC
FSC
NK1.1-PE
Single Cell Discrimination
FSC-H
FSC-A
Gate for TCRb+ Killer T Cells
CD8a
TCRb
Gate for Lymphocytes
SSC
FSC
TCRβ-APC
CD8α-PerCP-Cy5
Single Cell Discrimination
FSC-H
FSC-A
Gate for CD11b+
SSC
CD11b
Gate for KCs
Ly6C
F4/80
Gate for M1/M2 KCs
CD11c (M1)
CD206 (M2)
CD11c-APC
CD11b-PerCP-Cy5
Ly6C-FITC
F4/80-PE
CD206-Pacific Blue
M1
M2
CD4-APC
FoxP3-FITC
CD4-APC
FOXP3-FITC
IFNγ-PE
Supplemental Figure 1. Cell labeling, gating strategy, and identification of hepatic immune cells by flow cytometry.
